# Theoretical analysis of the dose dependence of the oxygen enhancement ratio and its relevance for clinical applications

**DOI:** 10.1186/1748-717X-6-171

**Published:** 2011-12-15

**Authors:** Tatiana Wenzl, Jan J Wilkens

**Affiliations:** 1 Department of Radiation Oncology, Technische Universität München, Klinikum rechts der Isar, Ismaninger Str. 22, 81675 Munich, Germany

**Keywords:** Oxygen enhancement ratio, hypoxia, oxygen effect, fractionation, high LET, radiation therapy

## Abstract

**Background:**

The increased resistance of hypoxic cells to ionizing radiation is usually believed to be the primary reason for treatment failure in tumors with oxygen-deficient areas. This oxygen effect can be expressed quantitatively by the oxygen enhancement ratio (OER). Here we investigate theoretically the dependence of the OER on the applied local dose for different types of ionizing irradiation and discuss its importance for clinical applications in radiotherapy for two scenarios: small dose variations during hypoxia-based dose painting and larger dose changes introduced by altered fractionation schemes.

**Methods:**

Using the widespread Alper-Howard-Flanders and standard linear-quadratic (LQ) models, OER calculations are performed for T1 human kidney and V79 Chinese hamster cells for various dose levels and various hypoxic oxygen partial pressures (pO2) between 0.01 and 20 mmHg as present in clinical situations *in vivo*. Our work comprises the analysis for both low linear energy transfer (LET) treatment with photons or protons and high-LET treatment with heavy ions. A detailed analysis of experimental data from the literature with respect to the dose dependence of the oxygen effect is performed, revealing controversial opinions whether the OER increases, decreases or stays constant with dose.

**Results:**

The behavior of the OER with dose per fraction depends primarily on the ratios of the LQ parameters alpha and beta under hypoxic and aerobic conditions, which themselves depend on LET, pO2 and the cell or tissue type. According to our calculations, the OER variations with dose *in vivo *for low-LET treatments are moderate, with changes in the OER up to 11% for dose painting (1 or 3 Gy per fraction compared to 2 Gy) and up to 22% in hyper-/hypofractionation (0.5 or 20 Gy per fraction compared to 2 Gy) for oxygen tensions between 0.2 and 20 mmHg typically measured clinically in hypoxic tumors. For extremely hypoxic cells (0.01 mmHg), the dose dependence of the OER becomes more pronounced (up to 36%). For high LET, OER variations up to 4% for the whole range of oxygen tensions between 0.01 and 20 mmHg were found, which were much smaller than for low LET.

**Conclusions:**

The formalism presented in this paper can be used for various tissue and radiation types to estimate OER variations with dose and help to decide in clinical practice whether some dose changes in dose painting or in fractionation can bring more benefit in terms of the OER in the treatment of a specific hypoxic tumor.

## Background

The poor treatment prognosis for tumors with oxygen-deficient areas is usually attributed to the decreased radiosensitivity of hypoxic cells. Hypoxia has been identified in many tumor types and the disadvantageous impact of hypoxia on local tumor control has been well recognized [1-4]. Due to the rapid development of noninvasive imaging methods to estimate the spatial distribution of the oxygen partial pressure within the tumor using different hypoxia markers [5-7], patient-specific treatment planning using dose painting or dose escalation in multifraction regimes based on functional hypoxia imaging is now considered a promising approach to overcome hypoxia in radiotherapy [8,9].

The radioprotective effect of hypoxia can be expressed quantitatively by the oxygen enhancement ratio (OER), which is defined as the ratio of doses *D(p_1_) *and *D(p_2_) *given under two different oxygenation conditions with oxygen partial pressures *p_1_*≤*p_2 _*to produce the same biological effect. Depending on the interest of the researcher, the comparison can be done between hypoxic-aerobic, anoxic-hypoxic, aerobic-hypobaric etc. conditions. Historically the OER has been defined as the radiation dose under anoxic conditions (*p_1 _*= 0 mmHg = const) divided by the dose under conditions of some partial pressure of oxygen *p_2 _= p*. In this way, the OER typically increases from unity at anoxic conditions to approximately 3 for normoxic conditions *in vitro*. Alternatively, OER is often stated as the ratio of the dose to hypoxic cells (at different levels of *p_1 _*= p) to the dose to aerobic cells (usually in air, *p_2 _*= 160 mmHg = const). In this case the OER decreases with increasing oxygen partial pressure in the cell environment, and the term hypoxia reduction factor (HRF) is sometimes used instead of OER here. In this paper, we employ the most general definition of OER with variable *p_1 _*and *p_2 _*at the same time.

It has been observed that the OER depends on many factors such as the oxygen partial pressures (pO_2_) for hypoxic and aerobic conditions, the tissue type, the linear energy transfer (LET) of the radiation and the chosen cell survival level (or alternatively the applied local dose). In this work we focus on the dose dependence of the OER and investigate if and how this dependence is important for clinical applications in radiotherapy. To open the discussion we review the experimental data of the dose dependence of the OER published from 1975 up to 2010. In contrary to the relative biological effectiveness (RBE), which was observed to decrease with increasing dose per fraction independent of radiation type and cell line, no clear tendency for the dose dependence of the OER (decreasing, increasing or constant) was reported in the literature.

The purpose of this paper is to estimate the amount of potential OER variations with dose in low- and high-LET radiotherapy and to assess the potential impact for clinical applications. Based on the previously developed OER model [10] and parameters for several tissue types, we will evaluate two clinically relevant scenarios: the first scenario deals with dose painting strategies, where an inhomogeneous dose within the target volume according to the oxygenation status is prescribed. Typically this involves relatively small local changes of the dose in the order of ± 50% of the mean dose per fraction. The second scenario considers much larger changes in the dose per fraction which occur if the overall fractionation scheme is modified (hyper- or hypofractionation).

## Methods

The survival of cells after exposure to a radiation dose is often described by the linear-quadratic (LQ) model [11]. This model is now in widespread use in both experimental and clinical radiobiology and generally works well in reproducing experimental results both *in vitro *and *in vivo*. We employed the standard LQ model with its two radiosensitivity parameters α and β in its most simplified form (without reoxygenation between fractions). Dose rate effects [12,13] were also not taken into account.

In this simplified case the fraction *S *of cells that survive an applied dose *D *may be written as:

(1) $$S=\exp\left(-\alpha D-\beta D^{2}\right)$$

The applied dose has to be changed to achieve the same biological effect under different irradiation conditions (e.g. high vs. low LET, aerobic vs. hypoxic environment etc.). This can be expressed by an enhancement factor (EF) defined as the ratio of doses given to a biological system under two different conditions, where the dose applied under the first condition 1 must be modified compared to the dose applied under the second condition 2 to obtain the same cell survival level:

(2) $$EF={\left(\frac{D_{1}}{D_{2}}\right|}_{S=const}$$

**Figure 1:**
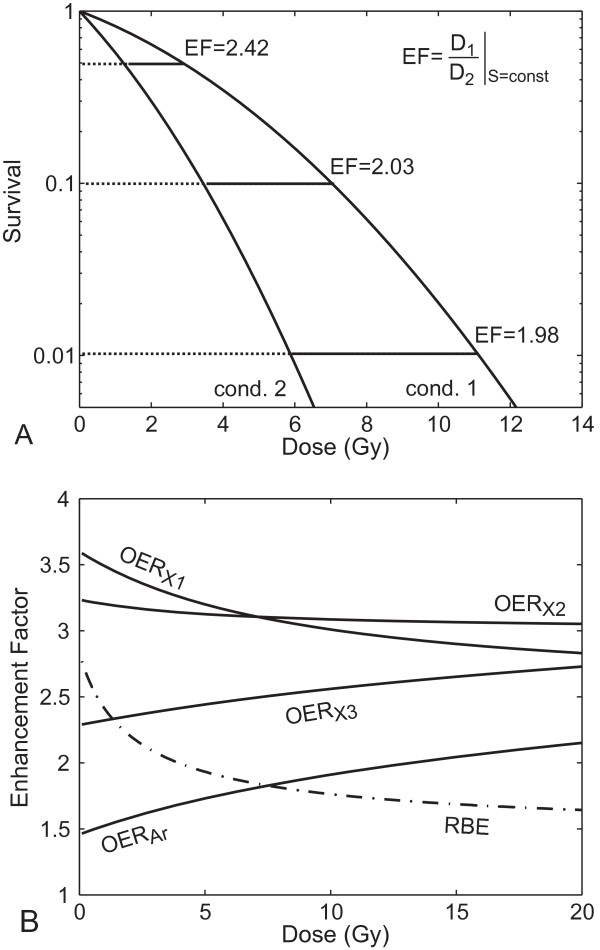
**Dose dependence of Survival and Enhancement Factor**. Panel A: Schematic illustration of the dependence of the enhancement factor (EF) on survival level and on dose. Panel B: Representative examples of the dose dependence of the EF: RBE (dashed-dotted line) and OER (solid lines) as a function of dose for high-LET radiation with argon ions (OER_Ar_) and low-LET radiation with x-rays (OER_X_) calculated using experimental data for V79 Chinese hamster cells *in vitro*. The OER curves were obtained using Eq. 6 and the experimental data for LQ parameters α*_a_*, α*_h_*, β*_a _*and β*_h _*from [26] (OER_Ar_, OER_X1_), [27] (OER_X2_) and [28] (OER_X3_). The RBE was calculated according to [29] using parameters α*_a _*and β*_a _*for argon ions and x-rays from [26].

Due to the nonlinear form of the initial shoulder of the survival curves, the EF can vary depending on the choice of the specific survival level used as endpoint. This means the EF depends on the applied local dose (Figure 1A). When comparing low- and high-LET radiation (e.g. x-rays and carbon ion beams), this enhancement factor is called RBE and Eq. 1 can be written as:

(3) $$RBE={\left(\frac{D_{x-rays}}{D_{carbon}}\right|}_{S=const}$$

If the same radiation type is compared under different oxygenation conditions (*p_1 _*and *p_2_*, with *p_1_*≤*p_2_*), this factor defines the OER:

(4) $$OER={\left(\frac{D\left(p_{1}\right)}{D\left(p_{2}\right)}\right|}_{S=const}$$

It was observed in the vast majority of experiments using various cell lines/tissue types and various radiation types that the RBE decreases with increasing dose per fraction (e.g. [14,15]), see Figure 1B for a typical example. The reason is that the x-ray survival curve usually has a relatively large initial shoulder, whereas the shoulder for high-LET radiation is usually smaller and the initial slope steeper (larger α value). For the OER, the situation is more complex. Depending on the cell line and the radiation type, some investigators found an increasing, some a decreasing and others a constant OER with increasing dose (see Figure 1B, and a list of relevant experimental studies in Tables 1 and 2), and the changes in the shape of the survival curves are quite variable. Table 2 adds experimental OER studies for high LET, which was here limited to the range 80-120 keV/μm.

**Table 1:** Summary of references to published mammalian cell survival data measured in experiments at low LET

**Ref**.	Cells	Rad. type (LET)	α*_a_*/α*_h_*	(β*_a_*/β*_h_*)^1/2^	OER(D)
[30]	V79-379A	x-rays	1.4	3.8	↗
[31]	V79-379A	x-rays	2.0	3.6	↗
[32]	V79-753B	x-rays	3.9	2.8	↘
[25]	V79-379A	x-rays			→
[33]	V79-B	x-rays			↗
[28]	V79-171	x-rays	2.3	3.6	↗
[26]	V79	protons (0.7 keV/μm)	1.7	4.5	↗
	V79	protons (1.9 keV/μm)	1.9	3.2	↗
	V79	x-rays	3.6	2.5	↘
[34]	V79	x-rays			↘
[27]	V79	x-rays	3.2	3.1	→
[33]	V79	x-rays			→
	CHO-6	x-rays			→
[35]	CHO-K1	^60^Co γ-rays			↗
	CHO-xrs6	^60^Co γ-rays			↗
[36]	CHO-K1	x-rays			↗
[37]	CHL-F	^60^Co γ-rays			→
[38]	R1	x-rays			→
[39]	R1	x-rays			→
[40]	FSa-II	^60^Co γ-rays	1.6	2.8	↗
	FSa-II	protons (1.9 keV/μm)	1.9	2.6	↗
[41]	T1	x-rays	5.0	2.3	↘
[33]	AA8	x-rays			↘
[42]	EMT6	^60^Co γ-rays			↗
[43]	B14 FAF28	^60^Co γ-rays			↘
[44]	U251	^60^Co γ-rays	4.0	2.5	↘

For each reference the cell line, the radiation type, the ratios of the radiosensitivity parameters (if provided) under aerobic (*a*) and hypoxic (*h*) conditions and the OER behavior as a function of dose (↗ increases, ↘ decreases or → remains nearly constant with increasing single dose) are given. The low-LET range was chosen between 0.2 and 2 keV/μm.

**Table 2:** Summary of references to published mammalian cell survival data measured in experiments at high LET

**Ref**.	Cells	Rad. type	LET (keV/μm)	α*_a_*/α*_h_*	(β*_a_*/β*_h_*)^1/2^	OER(D)
[26]	V79	argon	94	1.5	3.4	↗
[45]*	V79	carbon	102	2.7	1.1	↘
	V79	neon	110	2.0	1.3	↘
	HSG	carbon	88	2.2	1.3	↘
	HSG	neon	84	2.9	1.6	↘
[38]	R1	α-particles	110			→
[39]	R1	carbon	95			→
[46]	R1	carbon	90	2.0	1.6	↘
	R1	carbon	95	1.8	1.7	→
	R1	neon	90	1.4	1.9	↗
	R1	neon	120	1.7	1.5	→
	R1	argon	95	2.1	1.3	↘
[41]	T1	carbon	85	2.7	1.4	↘
	T1	neon	100	1.8	2.9	↗
	T1	argon	81	2.3	2.4	→
	T1	argon	91	2.0	2.5	↗
	T1	argon	117	1.7	1.7	→
[44]	U251	8 keV x-rays	> 50	1.3	> 2.9	↗

*Because of the large amount of the data in this reference for the LET range between 80 and 120 keV/μm, only a few representative cases are listed here to demonstrate the OER behavior.

For each reference the cell line, the ion beam type, the LET, the ratios of the radiosensitivity parameters (if provided) under aerobic (*a*) and hypoxic (*h*) conditions and the OER behavior as a function of dose (↗ increases, ↘ decreases or → remains nearly constant with increasing single dose) are given. The high-LET range was chosen between 80 and 120 keV/μm.

By equating the LQ-predicted surviving fractions (Eq. 1) for cells irradiated under aerobic (*a*) and hypoxic (*h*) conditions

(5) $$\begin{array}{c} S\left(D_{a}\right)=S\left(D_{h}\right)\\ \exp\left(-\alpha_{a}D_{a}-\beta_{a}D_{a}^{2}\right)=\exp\left(-\alpha_{h}D_{h}-\beta_{h}D_{h}^{2}\right) \end{array}$$

and using Eq. 4 one obtains a simple formula for the OER depending on dose *D_h _*given to hypoxic cells (which needs to be the dose per fraction in a multifraction regime) and on tissue specific parameters α*_a_*, α*_h_*, β*_a _*and β*_h_*:

(6) $$\begin{array}{l} OER(D_{h},\alpha_{a},\beta_{a},\alpha_{h},\beta_{h})=2D_{h}\beta_{a}/\\ \;\;\;\;\left(\sqrt{\alpha_{a}^{2}+4\beta_{a}(\alpha_{h}D_{h}+\beta_{h}D_{h}^{2})}-\alpha_{a}\right) \end{array}$$

In the limit of very small doses per fraction (*D_h_*→0), the OER is given by the ratio α*_a_*/α*_h_*, whereas for very large doses per fraction (*D_h_*→∞), OER is determined by the β parameters only:

(7) $$\begin{array}{c} OER\left(D_{h}\to 0\right)=\frac{\alpha_{a}}{\alpha_{h}}\\ OER\left(D_{h}\to \infty \right)=\sqrt{\frac{\beta_{a}}{\beta_{h}}} \end{array}$$

Since the first derivative of OER with respect to *D *never vanishes for *D *> 0 (unless α*_a_*/α*_h _*= (β*_a_*/β*_h_*)^1/2^), the OER increases with dose per fraction if the ratio α*_a_*/α*_h _*is smaller than (β*_a_*/β*_h_*)^1/2 ^for a specific cell line, and decreases with dose for α*_a_*/α*_h _*> (β*_a_*/β*_h_*)^1/2^. In the case of α*_a_*/α*_h _*= (β*_a_*/β*_h_*)^1/2^, the OER is independent of dose. In contrast to the RBE, where the ratio of the α values is typically much larger than the square root of the ratio of the β values (leading to a decreasing RBE with dose), these ratios show larger variability for the OER.

Obviously, the OER depends not only on dose and tissue type but also on LET and pO_2_. Based on Eq. 6 we assume that these dependences are determined by the LQ parameters α(*LET, pO_2_*) and β(*LET, pO_2_*). The following calculations were performed using an OER model that we presented previously [10]. Briefly, our model is based on the experimental data from the literature. For an analysis of these data in order to obtain the dependence of the LQ parameters α and β on pO_2 _we used (like many other investigators [6,16,17]) the concept of *Relative Radiosensitivity (RR) *according to the Alper and Howard-Flanders model [18]. The RR describes the response of a biological system to radiation dependent on oxygen tension *p *in the cell environment. RR is maximized at high oxygen concentrations and approaches unity for low oxygen levels:

(8) $$RR\left(p\right)=\frac{m\cdot p+1\cdot K}{p+K}$$

Here *m *is the maximum radiosensitivity and *K *is the oxygen concentration at which the RR equals half of its maximum. Based on this concept we assume an *Alper-Howard-Flanders dependence *of α(*p*) and β^1/2^(*p*) on pO_2_. These functions have a similar shape as the relative radiosensitivity, although the maxima and minima are different. Furthermore, we suggest in the clinically relevant LET region (where the RBE increases with LET) a simple linear dependence of α on LET and suppose β to be independent of LET (the dependence of β on LET was also discussed in [10]). Taking the pO_2 _and LET dependence together, one obtains:

(9) $$\begin{array}{c} \alpha \left(L,p\right)=\frac{\left(a_{1}+a_{2}\cdot L\right)\cdot p+\left(a_{3}+a_{4}\cdot L\right)\cdot K}{p+K}\\ \sqrt{\beta \left(L,p\right)}=\sqrt{\beta \left(p\right)}=\frac{b_{1}\cdot p+b_{2}\cdot K}{p+K} \end{array}$$

where *L *is LET and *a_1_, a_2_, a_3_, a_4_, b_1_, and b_2 _*are constant coefficients that were estimated by fitting the experimental data *in vitro *from the literature [10]. Finally, equation (6) can be written as:

(10) $$\begin{array}{c} OER\left(D_{h},L,p_{a},p_{h}\right)=2D_{h}\beta \left(L,p_{a}\right)/)\\ \left(\sqrt{\alpha^{2}\left(L,p_{a}\right)+4\beta \left(L,p_{a}\right)\left(\alpha \left(L,p_{h}\right)D_{h}+\beta \left(L,p_{h}\right)D_{h}^{2}\right)}-\alpha \left(L,p_{a}\right)\right) \end{array}$$

with oxygen partial pressures *p_a _*and *p_h _*under hypoxic and aerobic conditions (*p_h_*≤*p_a_*). As mentioned in **Background **these oxygen partial pressures are two independent values of the oxygen tension and the OER depends on the choice of both of them. Equation (10) can be used to describe the OER for different radiation types (low-LET and high-LET) and for various oxygen levels relevant for cell experiments *in vitro *and clinical situations *in vivo*. This OER model is a simple tool to quantify the oxygen effect in a practical way. The results of our model for the dependence of OER on LET and pO_2 _as discussed in our previous paper are in good agreement with preclinical and clinical studies [10].

In this paper we deal with the dependence of OER on dose per fraction for different irradiation types and degrees of hypoxia. The OER calculations were done for two cell types (V79 Chinese hamster cells and T1 human kidney cells) because only for these cell lines there was sufficient experimental data both in the low- and high-LET area (Tables 1 and 2). For low LET (0.2-2 keV/μm), the mean values of α and β found in the literature were taken (V79: α*_a _*= 0.135 Gy^-1^, β*_a _*= 0.032 Gy^-2^, α*_h _*= 0.06 Gy^-1^, β*_h _*= 0.003 Gy^-2^; T1: α*_a _*= 0.10 Gy^-1^, β*_a _*= 0.047 Gy^-2^, α*_h _*= 0.02 Gy^-1^, β*_h _*= 0.009 Gy^-2^). The values for V79 are similar to the data used by Carlson *et al*. [19] in an OER modeling study for prostate cancer and might therefore be relevant for clinical applications as well. The tissue parameters for high LET were estimated by an LET-dependent fitting of the aerobic and hypoxic experimental data *in vitro *for α and β in the full high-LET region between 10 and 260 keV/μm [10] to determine the parameters at 100 keV/μm (V79: α*_a _*= 0.75 Gy^-1^, β*_a _*= 0.061 Gy^-2^, α*_h _*= 0.41 Gy^-1^, β*_h _*= 0.014 Gy^-2^; T1: α*_a _*= 0.62 Gy^-1^, β*_a _*= 0.067 Gy^-2^, α*_h _*= 0.35 Gy^-1^, β*_h _*= 0.019 Gy^-2^).

## Results

**Figure 2:**
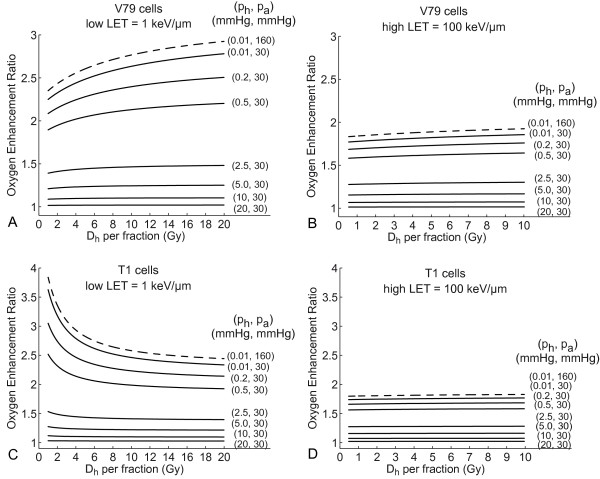
**Dose dependence of OER for V79 and T1 cells**. Dependence of OER on dose per fraction given to hypoxic cells for V79 (Panels A and B) and T1 cell lines (Panels C and D) at low-LET (Panels A and C) and high-LET (B and D). Solid lines show the model calculations for oxygen partial pressures *p_h _in vivo *between 0.01 and 20 mmHg (*p_a _*= 30 mmHg). Dashed lines correspond to cell experiments *in vitro *under extreme hypoxia (*p_h _*= 0.01 mmHg, *p_a _*= 160 mmHg).

OER values as a function of dose for V79 and T1 cells are shown in Figure 2 for low LET = 1 keV/μm (the middle of the range between 0.2 and 2 keV/μm) and high LET = 100 keV/μm (the middle of the range between 80 and 120 keV/μm). At low LET, OER is given for doses up to 20 Gy per fraction, whereas for high LET the physical absorbed dose is typically lower (due to the RBE), and the relevant dose is set up to 10 Gy per fraction. Of course, the predictions of the LQ model for high doses should be taken with great care. This model is well validated, both experimentally and theoretically, up to doses per fraction of about 10 Gy, and may be applicable to higher doses as well [20].

For V79 cells, the OER increases with dose at low and high LET, whereas it decreases with dose for T1 cells at low LET and stays almost constant for high LET. The dashed lines in Figure 2 show the OER for a typical cell experiment *in vitro *under extreme hypoxia (*p_h _*= 0.01 mmHg, *p_a _*= 160 mmHg). The ratios of the employed LQ parameters *in vitro *under hypoxic and aerobic conditions, which determine how and how strong the OER changes with increasing dose per fraction, were α*_a_*/α*_h _*= 2.25, (β*_a_*/β*_h_*)^1/2 ^= 3.27 (V79 cells) and α*_a_*/α*_h _*= 5.0, (β*_a_*/β*_h_*)^1/2 ^= 2.29 (T1 cells) at low LET and α*_a_*/α*_h _*= 1.83, (β*_a_*/β*_h_*)^1/2 ^= 2.09 (V79) and α*_a_*/α*_h _*= 1.77, (β*_a_*/β*_h_*)^1/2 ^= 1.88 (T1) at high LET. They can be compared with the ratios listed in Tables 1 and 2. If for example the value α*_a_*/α*_h _*for some experiment from Table 1 or 2 is lower than the value given above for V79 and the value (β*_a_*/β*_h_*)^1/2 ^higher as above for the same cell line and LET range, the curves of the dose dependence of OER will be steeper than in Figure 2 and the dose dependence is more pronounced.

To make the situation clinically more realistic, OER is also calculated for a set of hypoxic oxygen partial pressures *p_h _*between 0.2 and 20 mmHg, whereas *p_a _*= 30 mmHg is referred to as aerobic (well oxygenated cells *in vivo*) [15,21]. Since typical threshold values for the tumor oxygenation status in clinical and preclinical practice to estimate the treatment outcome of patients with a hypoxic tumor are < 2.5 mmHg, < 5 mmHg, < 10 mmHg and < 20 mmHg (see also Discussion in [10]), we calculated the dose dependence of the OER for these pO_2 _levels (solid lines in Figure 2). Because of the properties of clinically used Eppendorf histographs it is not possible to determine pO_2 _values for extreme hypoxia [22]. The lowest median pO_2 _values measured experimentally with Eppendorf histographs are typically 0.2 mmHg [6,23]. Figure 2 shows our calculation also for this oxygen level. Of course, cells at much lower oxygen concentrations can exist within a tumor and contribute to treatment failure. As a "worst case approximation", we also calculated the dependence of OER on dose for such extreme hypoxic cells *in vivo *(*p_h _*= 0.01 mmHg, *p_a _*= 30 mmHg). The OER values relevant for two scenarios of clinical dose variations are detailed in Table 3. For scenario 1 (dose painting), a mean dose per fraction of 2 Gy (low LET) or 1 Gy (high LET) is assumed as the baseline, and the OERs for ± 50% of this dose are given (which can be considered as extreme values for the dose variation within the target volume). For scenario 2 (hyper-/hypofractionation), the same baseline is used and OERs at one fourth (extreme hyperfractionation) and ten times the baseline (extreme hypofractionation) are evaluated. The values in Table 3 are calculated for a low, but still clinically measurable value of *p_h _*= 0.2 mmHg. The higher the oxygen partial pressure in a tumor, the less pronounced will be the variations of the OER with changing dose per fraction. For extremely hypoxic cells within a tumor, these variations could be more distinct (see Figure 2). For *p_h _*= 0.01 mmHg and *p_a _*= 30 mmHg, the changes in OER amount up to 6% (19%) for V79 und 26% (36%) for T1 cells if the dose per fraction is varied from 2 to 0.5 Gy (20 Gy) for low-LET radiation. For high LET, the changes in OER for extreme hypoxia are comparable with the changes at *p_h _*= 0.2 mmHg (Table 3).

**Table 3:** OER variations with dose per fraction

	**Low LET (1 keV/μm)**			**High LET (100 keV/μm)**		
		
	**Dose (Gy)**	**OER**		**Dose (Gy)**	**OER**	
				
		**V79**	**T1**		**V79**	**T1**
		
**Baseline**	2.0	2.16	2.74	1.0	1.70	1.66
						
**Scenario 1**	1.0	2.09 (-3%)	3.05 (+11%)	0.5	1.69 (-1%)	1.66 (0%)
(dose painting)	3.0	2.21 (+2%)	2.57 (-6%)	1.5	1.70 (0%)	1.66 (0%)
						
**Scenario 2**	0.5	2.05 (-5%)	3.32 (+21%)	0.25	1.69 (-1%)	1.66 (+0%)
(hyper-/hypo-fractionation)	20	2.52 (+17%)	2.14 (-22%)	10	1.77 (+4%)	1.68 (+1%)

The calculations were performed for V79 and T1 cell lines irradiated at *p_h _*= 0.2 mmHg with various dose levels. OER values (with respect to *p_a _*= 30 mmHg) are given for the baseline as well as for two scenarios of dose painting and hyper- or hypofractionation, along with the relative differences to the baseline situation (in percent).

## Discussion

In previous modelling studies regarding the OER [6,16,17,24,25], both dose-independent and dose-dependent implementations of the OER were used, mostly based on the Alper-Howard-Flanders formula of relative radiosensitivity (Eq. 8) or some modifications of this equation. With respect to experimental data published in the literature, decreasing, increasing and constant OERs with increasing dose for various cell lines and various radiation types were reported (Tables 1 and 2). Corresponding to our calculation in the framework of the LQ model, the OER depends on dose and its behavior is determined by the ratios of the LQ parameters α*_a_*/α*_h _*and (β*_a_*/β*_h_*)^1/2 ^under aerobic and hypoxic conditions (Eq. 7). Since these ratios can vary considerably with tissue/cell type, LET and pO_2 _(at least experimentally), this could explain the controversial findings from the publications in Tables 1 and 2. Only if these ratios are equal, the dose dependence disappears. This can be implemented on the modelling side if the pO_2 _dependence of α and β is given by α(*p*)= α_*a*_/*f*(*p*) and β(*p*) = β_*a*_/*f*(*p*)^2 ^(with the same function *f(p) *for both α and β) as used by Malinen *et al*. [6] or Carlson *et al*. [25]. One can argue whether or not this special case is actually realized in all cell lines or tissue types [25] (at least within experimental uncertainties), and whether a mechanistic interpretation of the LQ parameters and the underlying microscopic processes of radiation damage (see e.g. [12]) supports this situation. Given the caveats of mechanistic interpretations in radiation biology and the relatively large experimental error bars, a dose dependence of the OER can certainly not be excluded, which motivated us to study the potential clinical impact of a dose dependent OER.

The calculations performed in this work can be used to estimate - for a certain cell line or tissue type, irradiation type and oxygenation condition - whether, how and with which magnitude the OER varies with dose. Although the underlying OER model [10] used here was primarily based on experimental data from the literature *in vitro*, due to the implemented Alper-Howard-Flanders concept it can also provide reasonable predictions for preclinical and clinical situations *in vivo *(as discussed in detail in [10]). We therefore conclude that our analysis of the dependence of OER on dose per fraction has also some value for the assessment of realistic clinical situations, at least qualitatively.

Our investigation of the oxygen effect at clinically measured median oxygen tensions *p_h _*between 0.2 and 20 mmHg shows a relatively moderate dependence of the OER on dose per fraction (Table 3). The higher the oxygen partial pressure in a tumor, the less pronounced is the variation of the OER with dose (Figure 2). For hypoxia-based dose painting in the target volume (scenario 1, with local dose variations in the target between 1 and 3 Gy for low LET), the changes of the local OER *in vivo *were below ± 11% relative to the baseline of 2 Gy per fraction (Table 3). For high LET (dose variations between 0.5 and 1.5 Gy), these variations were much smaller (up to 1%). If the dose per fraction is varied over a larger range (by changing the fractionation scheme, scenario 2), OER variations in the order of ± 21% (0.5 Gy vs. 2 Gy per fraction) and ± 22% (20 Gy vs. 2 Gy per fraction) can be seen for low LET. Again, this was much smaller for high LET (up to 1% difference in OER for 0.25 Gy vs. 1 Gy and up to 4% for 10 Gy vs. 1 Gy per fraction). For a potential portion of extremely hypoxic cells within a tumor the dose dependence of the OER becomes more pronounced in low-LET treatment (see **Results**), but stays still moderate for high LET. One has to note that the values above depend strongly on the oxygenation conditions and tissue types, and the direction and magnitude of the OER variation could differ from the OERs for V79 and T1 cell lines. Nevertheless, the formalism presented in this paper can be used to estimate OER variations with dose and help to decide in clinical practice whether some changes in fractionation (hyper- or hypofractionation) can bring more benefit in the treatment of patients with a specific hypoxic tumor. For example, for human salivary gland (HSG) tumor cells the OER decreases with increasing dose under high-LET irradiation (Table 2). This means that a hypofractionated treatment in high-LET radiotherapy with heavy ions could be more advantageous with respect to the oxygen effect for patients with this kind of tumor. However, this can only be confirmed by clinical studies using high LET and various fractionation schemes, which (to the best of our knowledge) are currently not available for this tumor type. Alternatively, small animal experiments with implanted human tumors (which often exhibit large hypoxic fractions) could be a means to evaluate the impact of OER for various fractions schemes in a pre-clinical setting, and to validate the findings of our modeling study.

Another example can be based on the data published by Malinen *et al*. [6]. The authors investigated a spontaneous sarcoma of a dog with different hypoxic compounds within the tumor. In the most hypoxic area the measured average oxygen partial pressure *p_h _*was 0.2 mmHg. Using their data we calculated for this pO_2 _value α*_a_*/α*_h _*= 1.75 and (β*_a_*/β*_h_*)^1/2 ^= 3.25 (Eq. 4 and Table 1 in [6]). Compared with our analysis for V79 cell line under low-LET irradiation (α*_a_*/α*_h _*= 2.25 and (β*_a_*/β*_h_*)^1/2 ^= 3.27 and Figure 2A) the dependence of OER on dose per fraction for sarcomas from the study by Malinen *et al*. is also relatively moderate. The higher the oxygen partial pressure in a tumor, the less significant is the variation in the OER with the choice of dose per fraction. However, if hypoxia based treatment planning shall be performed, a larger number of fractions with smaller doses (hyperfractionation) could - due to the increasing OER with dose - bring more benefit with respect to the oxygen effect for the treatment of the sarcoma presented by Malinen *et al*. [6].

Although the OER seems to be only moderately dependent on the choice of dose per fraction, tumor hypoxia itself has a large negative effect on cell killing and there is potential for large errors in calculation of alternative dose fractionation schemas using models that do not account for tumor hypoxia at all [19], even if the dose dependence is not considered explicitly. In the case where measurements of oxygen partial pressures in a tumor are possible (e.g. with an Eppendorf histograph or noninvasive methods), the additional dose to hypoxic areas of the tumor required to achieve a constant biological effect in the whole target can be calculated in the framework of our model (Eq. 10) for various radiation types, oxygen tensions and tissue types. In the long run, this may help to overcome the adverse effects of low oxygen concentrations in many tumors.

## Conclusions

Since tumors with hypoxic areas exist and the treatment outcome of patients with hypoxic tumors is relatively poor, new predictive models are required to individualize and improve the treatment strategies for radiotherapy. In this work we investigated theoretically the importance of the dose dependence of the OER for clinical applications in radiotherapy. The analysis was performed for two scenarios: small dose variations within the target during dose painting and larger changes of the dose per fraction for different fractionation schemes. The calculations were performed for both low-LET treatment with photons or protons and high-LET treatment with heavy ions.

According to our analysis, the OER in clinical practice is moderately sensitive to the choice of dose per fraction. The OER can increase, decrease or remain a constant with increasing dose per fraction, and this behavior is determined by the ratios of the LQ parameters under hypoxic and aerobic conditions. These effects should be taken into account in hypoxia based treatment plan optimization. The formalism presented in this paper can be used to estimate OER variations with dose and to help with clinical decisions about any changes in dose prescription or treatment planning with respect to the oxygen effect. If simplified models without explicit consideration of the dose dependence are used for optimization in dose painting or changes of the fractionation scheme, our methods can be used to estimate the potential error in OER due to dose variations.

## List of abbreviations used

EF: Enhancement factor; HRF: Hypoxia reduction factor; LET: Linear energy transfer; LQ model: Linear-quadratic model; OER: Oxygen enhancement ratio; pO_2_: Oxygen partial pressure; RBE: Relative biological effectiveness; RR: Relative Radiosensitivity.

## Competing interests

The authors declare that they have no competing interests.

## Authors' contributions

All authors have made substantive intellectual contributions to the published research. Both TW and JJW have been involved in analyzing and interpreting the data from the literature, developing the model and preparation of the manuscript. All authors read and approved the final manuscript.
